# *MALAT1* long non-coding RNA is overexpressed in multiple myeloma and may serve as a marker to predict disease progression

**DOI:** 10.1186/1471-2407-14-809

**Published:** 2014-11-04

**Authors:** Shih-Feng Cho, Yuli Christine Chang, Chao-Sung Chang, Sheng-Fung Lin, Yi-Chang Liu, Hui-Hua Hsiao, Jan-Gowth Chang, Ta-Chih Liu

**Affiliations:** Graduate Institute of Clinical Medicine, College of Medicine, Kaohsiung Medical University, No.100, Shih-Chuan 1st Road, Kaohsiung, 807 Taiwan; Division of Hematology & Oncology, Department of Internal Medicine, Kaohsiung Medical University Hospital, Kaohsiung Medical University, No. 100, Tzyou 1st Road, Kaohsiung, 807 Taiwan; Department of Laboratory Medicine, Kaohsiung Medical University Hospital, No. 100, Tzyou 1st Road, Kaohsiung, 807 Taiwan; Graduate Institute of Healthcare Administration, Kaohsiung Medical University, No. 100, Shih-Chuan 1st Road, Kaohsiung, 807 Taiwan; Faculty of Medicine, College of Medicine, Kaohsiung Medical University, No. 100, Shih-Chuan 1st Road, Kaohsiung, 807 Taiwan; Epigenome Research Center, China Medical University Hospital, No. 2, Yuh-Der Road, Taichung, 404 Taiwan; Department of Laboratory Medicine, China Medical University Hospital, No. 2, Yuh-Der Road, Taichung, 404 Taiwan; School of Medicine, China Medical University, No.91, Hsueh-Shih Road, Taichung, 404 Taiwan

**Keywords:** Multiple myeloma, Long non-coding RNA, Metastasis-associated lung adenocarcinoma transcript 1 (*MALAT1*)

## Abstract

**Background:**

The pathogenesis of multiple myeloma involves complex genetic and epigenetic events. This study aimed to investigate the role and clinical relevance of the long non-coding RNA (lncRNA), metastasis-associated lung adenocarcinoma transcript 1 (*MALAT1*) in multiple myeloma.

**Methods:**

Bone marrow mononuclear cells were collected for analysis. The samples of multiple myeloma were taken from 45 patients at diagnosis, 61 post-treatment, and 18 who relapsed or had progression. Control samples were collected from 20 healthy individuals. Real-time quantitative reverse transcription polymerase chain reactions were performed to evaluate the expression of *MALAT1*. The clinical relevance of *MALAT1* expression was also explored.

**Results:**

*MALAT1* was overexpressed in the newly diagnosed patients compared with post-treatment patients (mean ∆C_T_: -5.54 ± 0.16 vs. -3.84 ± 0.09, 3.25-fold change; p < 0.001) and healthy individuals (mean ∆C_T_: -5.54 ± 0.16 vs. -3.95 ± 0.21, 3.01-fold change; p < 0.001). The expression of *MALAT1* strongly correlated with disease status, and the magnitude of change in *MALAT1* post-treatment had prognostic relevance. The patients with early progression had a significantly smaller change in *MALAT1* after treatment (mean ∆C_T_ change: 1.26 ± 1.06 vs. 2.09 ± 0.79, p = 0.011). A cut-off value of the change in *MALAT1* (∆C_T_ change: 1.5) was obtained, and the patients with a greater decrease in *MALAT1* (difference in ∆C_T_ >1.5) had significantly longer progression-free survival compared with the patients with a smaller *MALAT1* change (24 months vs. 11 months; p = 0.001). For the post-treatment patients, the risk of early progression could be predicted using this cut-off value.

**Conclusions:**

*MALAT1* was overexpressed in patients with myeloma and may play a role in its pathogenesis. In addition, *MALAT1* may serve as a molecular predictor of early progression.

**Electronic supplementary material:**

The online version of this article (doi:10.1186/1471-2407-14-809) contains supplementary material, which is available to authorized users.

## Background

Multiple myeloma is a hematological malignancy characterized by abnormal proliferation of monoclonal plasma cells in bone marrow leading to various end-organ damage including anemia, hypercalcemia, renal insufficiency and osteolytic bone disease [1]. The development of multiple myeloma is thought to result from monoclonal gammopathy of undetermined clinical significance [2, 3]. With the progression from monoclonal gammopathy of undetermined clinical significance to myeloma, several complex genetic events are involved including cytogenetic abnormalities, primary or secondary chromosomal translocation, and activation of oncogenes. These oncogenetic events include dysregulation of the *cyclin D* gene, mutation of *KRAS* or *NRAS*, and constitutively activated nuclear factor *κ*B (NF*κ*B) pathway [4–7]. In addition, the bone marrow microenvironment has also been reported to play an important role in the pathogenesis of this disease [8–10].

The human genome project revealed that at least 90% of the human genome is actively transcribed to RNA, but less than 2% of RNA encodes proteins [11, 12]. Non-coding RNAs (ncRNAs) are a class of RNA with little or no capacity for protein synthesis that includes small ncRNAs and long ncRNAs (lncRNAs), which have a length of more than 200 nucleotides. The lncRNAs have been highly conserved throughout mammalian evolution including in humans, and they have been shown to be aberrantly expressed in cancer tissue and to be involved in oncogenic or tumor suppressive processes [13].

Metastasis-associated lung adenocarcinoma transcript 1 (*MALAT1*) is one of the few biologically well-studied lncRNAs, and is located on chromosome 11 (11q13.1). This lncRNA is highly conserved in mammals and is more than 8000 nucleotides in length [14–16]. *MALAT1* has been shown to expressed in numerous tissues including the central nervous, endocrine, immune, reproductive and lymphoid systems [17, 18]. With respect to its function, *MALAT1* is localized to nuclear speckles and has been associated with regulation of gene expressions [19, 20]. In addition, *MALAT1* may play a role in the regulation of alternative splicing and cell cycle [21–23]. In terms of its association with cancer, *MALAT1* has been shown to be oncogenic and to be overexpressed in several solid tumors including lung, colorectal, bladder and laryngeal cancers [24–27].

The association between lncRNAs and multiple myeloma remains undetermined, and related studies are lacking. It has been reported that deregulation of the cell cycle is an important event during carcinogenesis, and that this event is also associated with *MALAT1* [23]. *MALAT1* has also been reported to be expressed broadly in human tissues including lymphoid tissues, bone marrow and B lymphocytes [28, 29]. Taken together, we hypothesized that *MALAT1* may play a role in multiple myeloma. Therefore, the aim of the present study was to evaluate the expression of *MALAT1* in bone marrow mononuclear cells from patients with multiple myeloma and with different disease status and healthy individuals.

## Methods

### Multiple myeloma patients and samples

The study cohort included adult patients (aged 20 years and older) with multiple myeloma diagnosed at Kaohsiung Medical University Hospital from 2007 to 2012 who were free from other coexisting malignant diseases. The diagnosis of multiple myeloma was confirmed by bone marrow analysis which revealed a monoclonal plasma cell count over 10% by definition and related laboratory tests. The patients of extramedullary myeloma were not enrolled to this study. The diagnostic criteria, disease status and response to treatment were based on the criteria of the International Myeloma Working Group [17–19]. Forty-five samples were collected from newly diagnosed patients (29 males, 16 females; median age 62.3 years, range 49 to 79 years) with different subtypes (IgG: 21, IgA: 13, light chain: 11) and clinical stages (Durie-Salmon stage 1: 1, stage 2: 6, stage 3: 38 or international staging system stage 1: 7, stage 2: 17, stage 3: 21). In addition, 61 samples were collected from patients after myeloma treatment, and 18 samples from patients who had experienced disease progression or relapse. The disease status of the post-treatment patients was mainly a complete response (CR) and very good partial response (VGPR) based on the criteria of International Myeloma Working Group. In addition, the percentage of plasma cells in the patients achieving VGPR or CR after treatment was less than 5%.

We also enrolled 20 healthy and genetically unrelated Taiwanese volunteers (healthy individuals) as the control group. These healthy individuals had undergone bone marrow analysis to investigate cytopenia that had been noted in blood tests, but whose bone marrow examinations revealed no abnormalities. All patients and healthy individuals signed informed consent forms after the study had been thoroughly explained.

The research protocol was created in accordance with the Declaration of Helsinki, and it was reviewed, approved and registered by the Ethics Committee of Kaohsiung Medical University Hospital (KMUHIRB-2012-01-08(II)).

### RNA extraction and reverse transcription

Bone marrow mononuclear cells were isolated for this study. First, the bone marrow samples were collected in tubes containing ethylenediaminetetraacetic acid (EDTA), preserved at 4°C and processed within 4 hours of collection. The bone marrow samples were then centrifuged at 12,000 × g for 15 minutes, after which ammonium chloride lysis buffer (10 mM NH4Cl, 10 mM KHCO3, 0.1 mM EDTA) was used to clear the red blood cells and effectively isolate the fraction of mononuclear cells.

The isolated bone marrow samples were stored at -80°C until RNA extraction. Isolation of RNA from 200 μL of cell suspension was carried out using the TRIzol protocol (Invitrogen). The extracted RNA was then treated with DNase (Promega) and the concentration was determined by spectrophotometric OD260 measurement. The integrity of the RNA was examined by 1.2% RNA denaturing agarose gel electrophoresis.

Reverse transcription was performed to generate complementary DNA in a final volume of 20 μL, containing 2 μg RNA, 25X dNTP mix (100 mM), 10X random primer (0.5 μM), RNase inhibiter, reverse transcriptase, reverse transcriptase buffer (10X) and diethylpyrocarbonate (DEPC)-treated water. The procedure was performed according to the manufacturer’s protocol (Applied Biosystems).

### Real-time quantitative reverse transcription polymerase chain reaction (RT-PCR) analysis of *MALAT1*expression

Real-time quantitative RT-PCR was performed in a final volume of 10 μL containing 1 μL of RT product, 0.6 μL of primer (Roche), 1.2 μL of probe (Roche, cat. no. 04688945001), 2.2 μL of DEPC H_2_O and 5 μL of qPCR Master Mix (2X) (KAPA Biosystem, KK 4600). Analysis of the human glyceraldehyde-3-phosphate dehydrogenase (*GAPDH*) gene was used as the internal control.

The primer sequences of *MALAT1* were as follows: forward, 5’-GACCCTTCACCCCTCACC-3’; reverse, 5’-TTATGGATCATGCCCACAAG-3’, and the primer sequences of *GAPDH* were as follows: forward, 5’-AAAGTCCGCCATTTTGCCACT-3’; and reverse, 5’-CCAAATCGTTAGCGCTCCTT-3’.

Real-time quantitative RT-PCR was performed in a LightCycler 480 Real-Time PCR System (Roche). The PCR cycling program consisted of incubation for enzyme activation at 95°C for 10 minutes, followed by melting at 95°C for 10 seconds, annealing at 60°C for 30 seconds, and then extension at 72°C for 1 second, for a total of 50 cycles.

The expression levels of *MALAT1* were normalized to the internal control *GAPDH* reference to obtain the relative threshold cycle (∆C_T_). The relative expression levels were calculated by the comparative C_T_ (∆∆C_T_) method, and relative expression folds (2^-ΔΔCT^) were calculated.

### Statistical analysis

The independent two samples t-test was used to compare the expression levels of *MALAT1* in the different subgroups. The frequency between each categorical variable was compared by the chi-square test (χ2 test), with Yates correction or Fisher’s exact test. Analysis of correlation was performed using Pearson correlations or Spearman correlation coefficients. Receiver operating characteristic (ROC) analysis was used to evaluate the cut-off value. Survival curves were plotted using the Kaplan–Meier method and compared using the log-rank test. Relative risk analysis was performed by calculating the odds ratio (OR) and 95% confidence interval (CI) by Cox regression analysis.

All statistical analyses were based on two-sided hypothesis tests with a significance level of p < 0.05. The analyses were performed using SPSS version 17.0 (SPSS, Chicago, IL, USA).

## Results

### Correlation of *MALAT1*expression with disease status in multiple myeloma

The expression of *MALAT1* was significantly higher in the patients at diagnosis compared with the patients post-treatment (mean ∆C_T_: -5.54 ± 0.16 vs. -3.84 ± 0.09, 3.25-fold change; p < 0.001) or the healthy individuals (mean ∆C_T_: -5.54 ± 0.16 vs. -3.95 ± 0.21, 3.01-fold change; p < 0.001) (Table 1). This suggests that *MALAT1* may be deregulated and overexpressed in patients with multiple myeloma.

**Table 1:** **Expression of** ***MALAT1*** **in patients with multiple myeloma and healthy individuals**

Population	No.	Expression of ***MALAT1***(Mean ΔC _T_)	
Newly diagnosed	45	-5.54 ± 0.16	
Post-treatment	61	-3.84 ± 0.09	
Relapse or progression	18	-4.92 ± 0.23	
Healthy individuals	20	-3.95 ± 0.21	
	ΔΔC_T_	Fold change	*P* value
Newly diagnosed vs. Post-treatment	-1.70	3.25	<0.001
Newly diagnosed vs. Healthy individuals	-1.59	3.01	<0.001
Relapse or progression vs. Post-treatment	-0.92	1.89	<0.001
Post-treatment vs. Healthy individuals	0.11	1.08	0.614

The 61 post-treatment samples were composed of 58 samples collected in disease status of VGPR or CR from 42 patients. The percentages of plasma cells were all less than 5%.

The 3 samples collected at a disease status of partial response came from 3 patients.

Note:

ΔC_T_ = C_T_ (*MALAT1* - *GAPDH*).

Increased expression (fold change) was calculated as 2^-ΔΔCT^.

**Figure 1:**
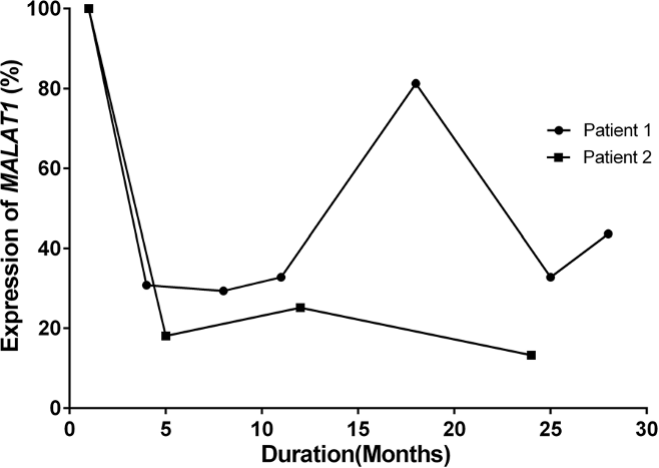
**Expression of** ***MALAT1*** **during treatment and follow-up in two representative patients.** The first patient (Patient 1) had a high expression of *MALAT1* initially, which then decreased after successful induction chemotherapy and autologous peripheral blood stem cell transplantation, 9 months after the time of diagnosis. At 18 months, disease relapse was accompanied by an increase in *MALAT1* expression. After salvage treatment, the disease was controlled and the expression of *MALAT1* decreased. Eventually, the disease progressed and the expression of *MALAT1* increased. The second patient (Patient 2) received induction chemotherapy followed by peripheral blood stem cell transplantation. The expression of *MALAT1* decreased markedly after the treatment achieved complete remission. The disease status remained in remission during the follow-up period and was accompanied by a low expression of *MALAT1*.

The association of *MALAT1* expression pattern with disease status was further analyzed. The expression of *MALAT1* was found to be significantly decreased in the post-treatment patients to a level that was similar to that of the healthy individuals (mean ∆C_T_: -3.84 ± 0.09 vs. -3.95 ± 0.21, p = 0.614). In addition, in the patients in whom the disease had progressed or relapsed, the expression of *MALAT1* was significantly increased compared with the post-treatment patients (mean ∆C_T_: -4.92 ± 0.23 vs. -3.84 ± 0.09, 1.89-fold change; p < 0.001) (Table 1). For the patients who underwent multiple bone marrow examinations during treatment and follow-up, the expression of *MALAT1* changed dynamically and was correlated with disease status (Figure 1).

### Association between *MALAT1*expression and clinical outcome

The clinical relevance of *MALAT1* was analyzed. The expressions of *MALAT1* in the 45 newly diagnosed patients with different clinical characteristics were listed in Additional file 1: Table S1. We noticed that the expression of *MALAT1* was not associated with the percentage of plasma cells in the bone marrow (r = -0.037, p = 0.808) (Additional file 2: Table S2). With regards to the association between *MALAT1* expression and prognosis, the results showed that the initial higher *MALAT1* expression level (Cut-off value: ∆C_T_ = -5.30) determined by ROC analysis was not associated with inferior prognosis including progression-free survival (PFS) (median PFS: 21.0 ± 9.9 vs. 15.0 ± 6.0 months, p = 0.390) or overall survival (OS) (median OS: Not reached; mean OS: 31.9 ± 4.3 vs. 37.8 ± 3.6 months, p = 0.172) (Additional file 3: Figure S1). However, we hypothesized the magnitude of the change (decrease) after myeloma-related therapy may have been related to the degree of treatment response and prognosis, because the expression of *MALAT1* changed after treatment.

Among the 45 patients, 36 (including 21 men) with a mean age of 61.3 years received bone marrow examinations at least twice including at diagnosis and after treatment, and hence the expression of *MALAT1* was available for further analysis. The median PFS of these 36 patients was 18 months (95% CI 8.95-25.35), and we used this median PFS as a cut-off value to divide the patients into two groups of early (PFS ≤18 months) or late (PFS >18 months) progression, with 18 patients in each group. We found that the only parameter which showed a significant difference between these two groups was the magnitude of *MALAT1* change after treatment. The patients with early progression had a significantly smaller magnitude of *MALAT1* change after treatment compared with the patients with late progression (mean difference of ∆C_T_: 1.26 ± 1.06 vs. 2.09 ± 0.79; p = 0.011) (Table 2).

**Table 2:** **The clinical characteristics of patients with early (PFS ≤18 months) or late (PFS >18 months) progression**

	All patients (N = 36)	PFS ≤ 18 months (N = 18)	PFS > 18 months (N = 18)	***P***value
Age (years, mean(SD))	61.3(8.3)	61.9 ± 7.8	60.6 ± 8.7	0.633
Male, n (%)	21(58.3%)	10(55.6%)	11(61.1%)	1.000
M protein				0.210
IgG, n (%)	19(52.8%)	7(38.9%)	12(66.6%)	
IgA, n (%)	10(27.8%)	7(38.9%)	3(16.7%)	
Light chain, n (%)	7(19.4%)	4(22.2%)	3(16.7%)	
International staging system				0.881
Stage 1, n (%)	5(13.9%)	2(11.1%)	3(16.7%)	
Stage 2, n (%)	12(33.3%)	6(33.3%)	6(33.3%)	
Stage 3, n (%)	19(52.8%)	10(55.6%)	9(50%)	
Durie-Salmon stage				1.000
Stage 1, n (%)	0	0	0	
Stage 2, n (%)	5(13.9%)	2(11.1%)	3(16.7%)	
Stage 3, n (%)	31(86.1%)	16(88.9%)	15(83.3%)	
Percentage of plasma cell in bone marrow (%, mean (SD))	50.8 ± 25.3	54.3 ± 26.8	47.3 ± 23.8	0.740
Anemia, n (%)	27(75%)	14(77.8%)	13(72.2%)	1.000
Renal insufficiency, n (%)	9(25%)	5(27.8%)	4(22.2%)	1.000
Hypercalcemia, n (%)	14(38.9%)	8(44.4%)	6(33.3%)	0.733
Bone disease, n (%)	25(69.4%)	14(77.8%)	11(61.1%)	0.471
Cytogenetic abnormality, n (%)	7(19.4%)	3(16.7%)	4(22.2%)	1.000
Bortezomib-containing induction Tx, n (%)	11(30.6%)	4(22.2%)	7(38.9%)	0.471
Auto-HSCT in 1st fine Tx, n (%)	9(25%)	2(11.1%)	7(38.9%)	0.121
Treatment response:				
CR, n (%)	7(19.4%)	1(5.6%)	6(33.3%)	0.088
VGPR, n (%)	26(72.2%)	14(77.8%)	12(66.7%)	0.711
PR, n (%)	3(8.3%)	3(16.7%)	0	0.229
Expression of *MALAT1* at diagnosis (Mean ΔC_T_ ± SD)		-5.52 ± 1.15	-5.77 ± 0.89	0.353
Magnitude of *MALAT1* change after treatment (Difference in ΔC_T_)		1.26 ± 1.06	2.09 ± 0.79	0.011

Difference in ΔC_T_ = ΔC_T_ (Post-treatment - newly diagnosed).

Auto-HSCT, autologous hematopoietic stem cell transplantation; CR, complete response; VGPR, very good partial response; PFS, progression-free survival; Tx, treatment.

### Role of *MALAT1*in predicting early progression

The previous results showed that the magnitude of *MALAT1* change (as quantified by the difference in ∆C_T_) was the only parameter associated with PFS. We then used ROC analysis and obtained a cut-off expression change value of 1.5 (post-treatment ∆C_T_ – pre-treatment ∆C_T_; approximately a 2.8-fold change) with an estimated area under the ROC curve of 0.79 (p = 0.003). The proportion of patients with a lower *MALAT1* change (difference in ∆C_T_ ≤1.5) was significantly higher in those who displayed early progression (n = 13, 72.2%) compared with those who displayed late progression (n = 4, 22.2%; p = 0.007).

**Figure 2:**
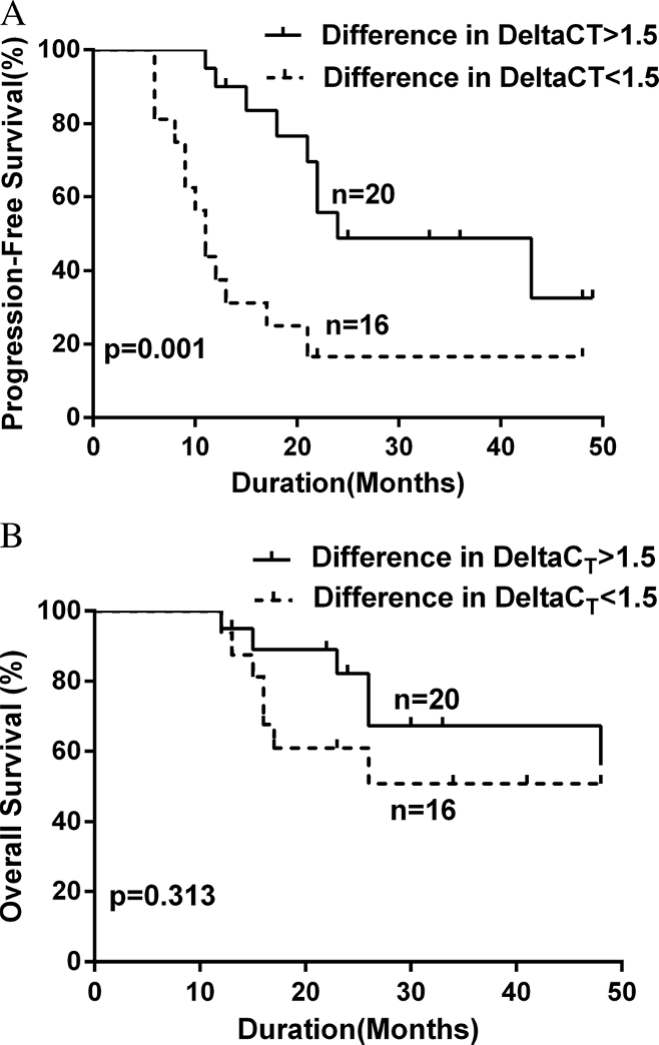
**Kaplan-Meier estimates of the probability of progression-free survival (PFS, A) and overall survival (OS, B) are shown according to the magnitude in the change of** ***MALAT1*** **expression after treatment.** The patients were divided into two groups by a cut-off value (difference in ΔC_T_: 1.5).

In terms of an association between clinical characteristics and magnitude of *MALAT1* change, the patients with greater changes (difference in ∆C_T_ >1.5) may have had a better treatment response (Additional file 4: Table S3). The PFS and OS rates were also analyzed using the cut-off value from ROC analysis. With a minimum follow-up period of 12 months (range: 12 to 48 months), the patients with a greater *MALAT1* decrease (difference in ∆C_T_ >1.5) had a significantly prolonged median PFS (24 months, range 11-48 months) compared with the patients with a smaller *MALAT1* change (11 months, range: 6-21 months; p = 0.001). There was no significant difference in OS between the two groups (median OS: Not reached; mean OS: 39.2 ± 3.6 months, range: 12-48 months vs. 32.8 ± 4.2 months, range: 12-48 months; p = 0.313) (Figure 2).

Cox regression analysis was used to identify the relative risk of early progression, which revealed that autologous hematopoietic stem cell transplantation (Auto-HSCT) and the magnitude of *MALAT1* change were significantly associated with the prognosis. For all post-treatment patients (n = 36), those with a smaller *MALAT1* change (difference in ∆C_T_ ≤1.5) had a significantly higher risk of early progression of disease (OR 4.89, 95% CI 1.73-13.86; p = 0.003), while auto-HSCT reduced the risk of early progression (OR 0.22, 95% CI 0.05-0.97; p = 0.046). For the post-treatment patients with a VGPR or CR (n = 33), a smaller *MALAT1* change (difference in ∆C_T_ ≤1.5) remained the single factor predictive of early progression of multiple myeloma (OR 4.38, 95% CI 1.48-12.99; p = 0.008) (Table 3). Using the cut-off value to predict the patients who would show early progression (PFS ≤18 months), the estimated accuracy was 75% with a sensitivity of 72.2%, a specificity of 77.8%, a positive predictive value of 76.5%, and a negative predictive value of 73.7%.

**Table 3:** **Multivariate Cox regression analysis for all post-treatment patients and post-treatment patients with a treatment response of VGPR/CR**

			PFS ≤18 months (N = 18)	PFS >18 months (N = 18)	Cox regression analysis		
					OR	95% CI	***P***value
All patients (N = 36)	Auto-HSCT in 1st line treatment, n	9	2(11.1%)	7(38.9%)	0.22	0.05-0.97	0.046
	Difference in △C_T_ ≤1.5, n	17	13(72.2%)	4(22.2%)	4.89	1.73-13.86	0.003
			PFS ≤18 months (N = 15)	PFS >18 months (N = 18)			
Patients with VGPR/CR (N = 33)	Auto-HSCT in 1st line treatment, n	9	2(13.3%)	7(38.9%)	0.24	0.05-1.09	0.066
	Difference in △C_T_ ≤1.5, n	14	10(66.7%)	4(22.2%)	4.38	1.48-12.99	0.008

OR, Odds ratio; CI, confidential interval; Auto-HSCT, autologous hematopoietic stem-cell transplantation; CR, complete response; VGPR, very good partial response; PFS, progression-free survival.

## Discussion

In the current study, we demonstrated that *MALAT1* was overexpressed in the patients with newly diagnosed multiple myeloma. This finding indicates that *MALAT1* may play a role in multiple myeloma.

The results of the present study are in contrast with the study by Isin et al., in which the expression of *MALAT1* was found to be significantly lower in patients with multiple myeloma [30]. A possible explanation for this discrepancy may be due to different sample sources. Our study analyzed the expression of *MALAT1* in bone marrow mononuclear cells rather than plasma samples, because the pathogenesis of myeloma is closely related to bone marrow. Another possible explanation for the higher expression of *MALAT1* in the current study may be associated with the bone marrow microenvironment which supports the proliferation of myeloma cells. In addition, our analysis revealed that expression of *MALAT1* in newly diagnosed myeloma patients is not associated with the total percentage of plasma cells in the bone marrow. This finding indicated that the expression of *MALAT1* may be associated with interactions between myeloma cells and the bone marrow microenvironment. The detailed mechanism needs further studies to elucidate.

The current study also investigated the clinical relevance of *MALAT1* in patients with multiple myeloma. We found that the expression of *MALAT1* changed dynamically when stratified by disease status. In addition, the major clinical significance was the magnitude of change in expression after treatment rather than the initial expression. This finding is different from previous studies of solid tumors which have reported that a higher expression is related to poorer prognosis. We observed that the patients with a greater decrease in *MALAT1* after initial treatment had a significantly prolonged PFS, which is consistent with the current consensus that therapeutic intervention to achieve a maximal response is beneficial for patients with multiple myeloma [31, 32]. In terms of OS, we did not find a significant benefit in the post-treatment patients with a greater decrease in *MALAT1*. A possible explanation may be the incorporation of potent and effective salvage treatment in the patients who experienced a relapse or progression of disease, as well as the fact that some patients received auto-HSCT after salvage treatment.

We also found that *MALAT1* may serve as a marker to predict early progression. Because the duration of response decreases with an increasing number of salvage regimens after progression, identification of patients at risk of early progression after first-line treatment is an important issue. More intensive treatment may improve the prognosis in this subgroup. We also found that patients with a smaller *MALAT1* change after treatment had a significantly higher risk for early progression, even in those with a VGPR, CR and normal percentage of plasma cells in bone marrow. This finding suggests that the expression of *MALAT1* can be used to identify the patients at risk of early progression. Accordingly, the therapeutic strategy may be adjusted to be initially more aggressive, as more potent treatment may reduce the risk of early progression and prolong PFS.

Our findings may provide a new insight into the pathogenesis of multiple myeloma. However, there are some limitations to this study. First, the cytogenetic examinations were done by conventional G-band metaphase chromosome analysis, and the percentage of cytogenetic abnormalities was relative low. Therefore, the association between *MALAT1* and specific cytogenetic abnormalities remains to be determined. Further analysis by fluorescent in-situ hybridization with larger cohort may provide more impactful insight on the clinical relevance of *MALAT1* expression in multiple myeloma. Second, we didn’t evaluate the expression of *MALAT1* in patients resistant to myeloma therapy due to no available samples. Third, the number of cases to evaluate the clinical relevance of *MALAT1* was limited, which was likely due to the stringency of the enrollment criteria.

## Conclusions

In conclusion, this study revealed that *MALAT1* was overexpressed in patients with multiple myeloma, and this lncRNA may play a role in the pathogenesis of the disease. In addition, the change in *MALAT1* expression after treatment was clinically significant and may serve as a molecular predictor of the patients at risk of early progression of multiple myeloma.

## Electronic supplementary material

Additional file 1: Table S1: The clinical characteristics and expression of *MALAT1* in 45 newly diagnosed patients with multiple myeloma. (DOC 56 KB)

Additional file 2: Table S2: Expression of *MALAT1* and plasma cell percentage in the bone marrow in 45 newly diagnosed patients. (DOC 58 KB)

Additional file 3: Figure S1: Kaplan-Meier estimates of the probability of progression-free survival (PFS, 1A) and overall survival (OS, 1B) are based on the initial level of *MALAT1* expression. The patients were divided into two groups by a cut-off value (△C_T_: -5.30). (ZIP 137 KB)

Additional file 4: Table S3: The clinical characteristics of the patients with different cut-off values. (DOC 58 KB)
