# Supplementary material for: MALAT1 long non-coding RNA is overexpressed in multiple myeloma and may serve as a marker to predict disease progression
Source: BMC Cancer. 2014 Nov 4;14:809. doi: 10.1186/1471-2407-14-809 (PMC4233101; doi:10.1186/1471-2407-14-809)
Supplement: Supplementary file 1 — Additional file 1: Table S1: The clinical characteristics and expression of MALAT1 in 45 newly diagnosed patients with multiple myeloma. (DOC 56 KB) [file 12885_2014_4995_MOESM1_ESM.doc]

Supplemental Table 1. The clinical characteristics and expression of *MALAT1* in 45 newly diagnosed patients with multiple myeloma.

|  | **No.** | **Expression of *MALAT1***  **(Mean** Δ**C_T_)** | ***P*-value** |
| --- | --- | --- | --- |
| **Gender** |  |  |  |
| Male, n (%) | 29(64.4%) | -5.50±0.93 | 0.352 |
| Female, n (%) | 16(35.6%) | -5.89±1.10 |  |
| **Age (years)** |  |  |  |
| >60, n (%) | 25(55.5%) | -5.69±1.09 | 0.762 |
| ≤60, n (%) | 20(44.5%) | -5.50±0.99 |  |
| **M protein** |  |  |  |
| IgG, n (%) | 21(46.7%) | -5.59±1.02 | 0.833 |
| IgA, n (%) | 13(28.9%) | -5.57±1.16 |  |
| Light chain, n (%) | 11(24.4%) | -5.81±0.96 |  |
| **ISS stage** |  |  |  |
| I, n (%) | 7(15.6%) | -5.63±0.90 | 0.606 |
| II, n (%) | 17(37.8%) | -5.84±1.06 |  |
| III, n (%) | 21(46.6%) | -5.51±1.02 |  |
| **Durie-Salmon stage** |  |  |  |
| I, n (%) | 1(2.2%) | -6.62 | 0.407 |
| II, n (%) | 6(13.3%) | -5.22±0.53 |  |
| III, n (%) | 38(84.5%) | -5.68±1.06 |  |
| **Hypercalcemia** |  |  |  |
| With, n (%) | 16(35.5%) | -5.56±1.13 | 0.707 |
| Without, n (%) | 29(64.5%) | -5.63±1.06 |  |
| **Renal insufficiency** |  |  |  |
| With, n (%) | 12(26.7%) | -5.56±1.03 | 0.479 |
| Without, n (%) | 33(73.3%) | -5.62±1.06 |  |
| **Anemia** |  |  |  |
| With, n (%) | 32(71.1%) | -5.64±1.02 | 0.865 |
| Without, n (%) | 13(28.9%) | -5.51±1.74 |  |
| **Bone disease** |  |  |  |
| With, n (%) | 30(66.7%) | -5.44±0.96 | 0.410 |
| Without, n (%) | 15(33.3%) | -5.93±1.16 |  |
| **Cytogenetic abnormality** |  |  |  |
| With, n (%) | 9(20%) | -5.37±1.01 | 0.535 |
| Without, n (%) | 36(80%) | -5.66±1.05 |  |

Pooled t-test and ANOVA test for continuous variables.
