# Supplementary material for: MALAT1 long non-coding RNA is overexpressed in multiple myeloma and may serve as a marker to predict disease progression
Source: BMC Cancer. 2014 Nov 4;14:809. doi: 10.1186/1471-2407-14-809 (PMC4233101; doi:10.1186/1471-2407-14-809)
Supplement: Supplementary file 2 — Additional file 2: Table S2: Expression of MALAT1 and plasma cell percentage in the bone marrow in 45 newly diagnosed patients. (DOC 58 KB) [file 12885_2014_4995_MOESM2_ESM.doc]

| No. of patient | % of PC | Expression of *MALAT1* | No. of patient | % of PC | Expression of *MALAT1* |
| --- | --- | --- | --- | --- | --- |
| 1 | 68 | -5.44 | 25 | 13 | -4.51 |
| 2 | 82.6 | -5.65 | 26 | 50 | -6.05 |
| 3 | 70 | -5.26 | 27 | 50 | -5.15 |
| 4 | 19 | -6.35 | 28 | 78 | -5.65 |
| 5 | 90 | -5.02 | 29 | 36 | -7.68 |
| 6 | 66.8 | -7.04 | 30 | 23 | -4.58 |
| 7 | 90 | -4.96 | 31 | 19 | -5.59 |
| 8 | 66.6 | -3.94 | 32 | 33.1 | -6.11 |
| 9 | 50 | -6.07 | 33 | 57.7 | -4.63 |
| 10 | 83.2 | -6.65 | 34 | 50 | -3.77 |
| 11 | 12.5 | -6.72 | 35 | 60 | -7.13 |
| 12 | 66.8 | -5.17 | 36 | 11.4 | -6.15 |
| 13 | 51 | -4.97 | 37 | 25 | -6.62 |
| 14 | 64 | -6.8 | 38 | 88 | -6.65 |
| 15 | 15 | -4.6 | 39 | 64.2 | -5.68 |
| 16 | 47.5 | -4.58 | 40 | 40.4 | -5.39 |
| 17 | 39 | -7.8 | 41 | 25 | -4.94 |
| 18 | 69.2 | -5.28 | 42 | 85 | -7.8 |
| 19 | 95 | -4.46 | 43 | 81.2 | -5.59 |
| 20 | 11.2 | -5.33 | 44 | 65 | -6.65 |
| 21 | 90 | -5.03 | 45 | 95.2 | -5.86 |
| 22 | 26.5 | -4.11 |  |  |  |
| 23 | 44 | -5.02 |  |  |  |
| 24 | 56.6 | -3.97 |  |  |  |

Supplemental Table 2. Expression of *MALAT1* and plasma cell percentage in the bone marrow in 45 newly diagnosed patients.

Expression of MALAT1 was presented as ΔC_T_ value.

PC, plasma cell
