# Supplementary material for: MALAT1 long non-coding RNA is overexpressed in multiple myeloma and may serve as a marker to predict disease progression
Source: BMC Cancer. 2014 Nov 4;14:809. doi: 10.1186/1471-2407-14-809 (PMC4233101; doi:10.1186/1471-2407-14-809)
Supplement: Supplementary file 4 — Additional file 4: Table S3: The clinical characteristics of the patients with different cut-off values. (DOC 58 KB) [file 12885_2014_4995_MOESM4_ESM.doc]

Supplemental Table 3. The clinical characteristics of the patients with different cut-off values.

|  | All patients  (N=36) | Difference in  ΔC_T_ >1.5  (N= 19) | Difference in  ΔC_T_ ≤1.5  (N= 17) | *P* value |
| --- | --- | --- | --- | --- |
|
| Age (years, mean(SD)) |  | 62.8(8.1) | 59.5(8.2) | 0.216 |
| Male, n (%) | 21(58.3%) | 11(57.9%) | 10(58.8%) | 1.000 |
| M protein |  |  |  | 0.416 |
| IgG, n (%) | 19(52.8%) | 12(63.2%) | 7(41.2%) |  |
| IgA, n (%) | 10(27.8%) | 4(21.1%) | 6(35.3%) |  |
| Light chain, n (%) | 7(19.4) | 3(15.7%) | 4(23.5%) |  |
| International staging system |  |  |  | 0.286 |
| Stage 1, n (%) | 5(13.9%) | 4(21.1%) | 1(5.9%) |  |
| Stage 2, n (%) | 12(33.3%) | 7(36.8%) | 5(29.4%) |  |
| Stage 3, n (%) | 19(52.8%) | 8(42.1%) | 11(64.7%) |  |
| Durie-Salmon stage |  |  |  | 1.000 |
| Stage 1, n (%) | 0 | 0 | 0 |  |
| Stage 2, n (%) | 5(13.9%) | 3(15.8%) | 2(11.8%) |  |
| Stage 3, n (%) | 31(86.1%) | 16(84.2%) | 15(88.2%) |  |
| Percentage of plasma cell in bone marrow (%,mean(SD)) | 50.8±25.3 | 51.7±25.7 | 49.7±25.5 | 0.240 |
| Anemia, n (%) | 27(75%) | 14(73.7%) | 13(76.5%) | 1.000 |
| Renal insufficiency, n (%) | 9(25%) | 5(26.3%) | 4(23.5%) | 1.000 |
| Hypercalcemia, n (%) | 14(38.9%) | 7(36.8%) | 7(41.2%) | 1.000 |
| Bone disease, n (%) | 25(69.4%) | 11(57.9%) | 14(82.4%) | 0.156 |
| Cytogenetic abnormality, n (%) | 7(19.4%) | 2(10.5%) | 5(29.4%) | 0.219 |
| Bortezomib-containing induction Tx, n (%) | 11(30.6%) | 7(36.8%) | 4(23.5%) | 0.481 |
| Auto-HSCT in 1st fine Tx, n (%) | 9(25%) | 6(31.6%) | 3(17.6) | 0.451 |
| Treatment Response: |  |  |  |  |
| CR, n (%) | 7(19.4%) | 6(31.6%) | 1(5.9%) | 0.092 |
| VGPR, n (%) | 26(72.2%) | 13(68.4%) | 13(76.5%) | 0.717 |
| PR, n (%) | 3(8.3%) | 0 | 3(17.6%) | 0.095 |
| Expression of *MALAT1* at diagnosis (Mean ΔC_T_ ± SD) |  | -5.77±1.04 | -5.36±1.03 | 0.281 |

Difference in ΔC_T_ = ΔC_T_ (Post-treatment - newly diagnosed)

Auto-HSCT, autologous hematopoietic stem-cell transplantation; CR, complete response; VGPR, very good partial response; PFS, progression-free survival; Tx, treatment
